
JOURNAL INFORMATION
==============================
NLM Title Abbreviation: Medizinrecht
Journal ID: Medizinrecht
NLM Journal ID: 8309354

Medizinrecht

ISSN: 0723-8886
EISSN: 1433-8629

ARTICLE INFORMATION
==============================
PMCID: PMC9778451
PMID: 36575716
DOI: 10.1007/s00350-022-6357-9
Article ID: 6357
Article version: 1
Subjects: Aufsätze

Kein Malus für Ungeimpfte!

Rechtliche Einwände gegen finanzielle Malusregelungen für nicht gegen Covid-19 geimpfte Personen

Lauppert von Peharnik Christina 1
Freiherr von Ulmenstein Ulrich 1
Tretter Max 2
1 grid.8664.c 0000 0001 2165 8627 Justus-Liebig-Universität Gießen, Hein-Heckroth-Straße 5, 35390 Gießen, Deutschland
2 grid.5330.5 0000 0001 2107 3311 Lehrstuhl für Systematische Theologie II (Ethik), Friedrich-Alexander-Universität Erlangen-Nürnberg, Kochstraße 6, 91054 Erlangen, Deutschland
Electronic publication date: 2022 Dec 22
Print publication date: 2022
Volume: 40
Issue: 12
First page: 1005
Last page: 1010
Copyright: © Der/die Autor(en) 2022
License: Open Access Dieser Artikel wird unter der Creative Commons Namensnennung 4.0 International Lizenz veröffentlicht, welche die Nutzung, Vervielfältigung, Bearbeitung, Verbreitung und Wiedergabe in jeglichem Medium und Format erlaubt, sofern Sie den/die ursprünglichen Autor(en) und die Quelle ordnungsgemäß nennen, einen Link zur Creative Commons Lizenz beifügen und angeben, ob Änderungen vorgenommen wurden.
Die in diesem Artikel enthaltenen Bilder und sonstiges Drittmaterial unterliegen ebenfalls der genannten Creative Commons Lizenz, sofern sich aus der Abbildungslegende nichts anderes ergibt. Sofern das betreffende Material nicht unter der genannten Creative Commons Lizenz steht und die betreffende Handlung nicht nach gesetzlichen Vorschriften erlaubt ist, ist für die oben aufgeführten Weiterverwendungen des Materials die Einwilligung des jeweiligen Rechteinhabers einzuholen.
Weitere Details zur Lizenz entnehmen Sie bitte der Lizenzinformation auf http://creativecommons.org/licenses/by/4.0/deed.de.
License URL: https://creativecommons.org/licenses/by/4.0/

issue-copyright-statement: © C.H. Beck Verlag oHG, München und Springer-Verlag GmbH Deutschland, ein Teil von Springer Nature 2022

==============================

**Christina Lauppert von Peharnik, Ulrich Freiherr von Ulmenstein und Max Tretter***

Seit über zwei Jahren stellt die Covid-19-Pandemie die Gesellschaft vor immer neue Herausforderungen und Fragestellungen. Im Spannungsfeld von Solidarität und Eigenverantwortung greift der Beitrag die Frage auf, ob Malusregelungen für nicht gegen Covid-19 geimpfte Personen nach geltendem Recht realisierbar sind und ob sie unter Zugrundelegung verfassungsrechtlicher Maßstäbe künftig normiert werden könnten.

Zunächst werden die *de lege lata* bestehenden Möglichkeiten der Individuellen Beitragserhöhung und der ganz oder teilweisen Beteiligung an Krankheitskosten und der ganz oder teilweisen Rückforderung des Krankengeldes erörtert.

Hierauf folgt die Betrachtung *de lege ferenda*, die die verfassungsrechtlichen Erwägungen enthält und die Entwicklung gesamtgesellschaftlich und gesundheitspolitisch einordnet.

### I. Einleitung

Zu Beginn der Covid-19-Pandemie vor über zwei Jahren war das Risiko (schwer) an Covid-19 zu erkranken zunächst nicht quantifizierbar. Unklar war auch, wie lange es dauern würde einen Impfstoff zu entwickeln und wie wirksam dieser sein würde. Die große Hoffnung war, dass die Impfstoffentwicklung zügig gelänge und die Wirksamkeit möglichst hoch wäre, sodass der Pandemie schnell ein Ende gesetzt werden könnte. Heute kennt man die Ansteckungsraten bei verschiedenen Aktivitäten und die Risikofaktoren für schwere Verläufe. Impfstoffe wurden entwickelt und in einem beispiellos schnellen und unbürokratischen Verfahren zugelassen. Auch die Wirksamkeit gegenüber den anfänglichen Alpha-Varianten war – im Gegensatz zur damaligen Impfquote – hoch. Denn womit seitens der Politik scheinbar nicht gerechnet wurde, ist die Impfskepsis eines Teils der Bevölkerung. Zur Überwindung dieser Impfskepsis wurde die Einführung einer Allgemeinen Impfpflicht diskutiert und letzten Endes eine bereichsbezogene Impfpflicht etabliert[2]. Um weiteren Druck auf ungeimpfte Personen auszuüben, wurde Ende 2021 von verschiedenen Politikern, unter anderem dem Bayerischen Gesundheitsminister *Klaus Holetschek* und dem Tübinger Oberbürgermeister *Boris Palmer*, vorgeschlagen, die Versicherten, die sich nicht impfen lassen, obwohl sie es aus medizinischer Sicht könnten, mit Malusregelungen zu belegen[3]. Unter Malusregelungen werden dabei vielgestaltige Formen einer finanziellen (Mit-)Beteiligung Covid-19-ungeimpfter Personen an den statistisch von ihnen verursachten Mehrkosten verstanden. Diese reichen von

Christina Lauppert von Peharnik (Ass. iur.),
Wissenschaftliche Mitarbeiterin in der
Professur für Öffentliches Recht, Justus-Liebig-Universität Gießen,
Hein-Heckroth-Straße 5, 35390 Gießen, Deutschland

Ulrich Freiherr von Ulmenstein (Dipl. iur.),
Wissenschaftlicher Mitarbeiter in der
Professur für Öffentliches Recht, Justus-Liebig-Universität Gießen,
Hein-Heckroth-Straße 5, 35390 Gießen, Deutschland

Max Tretter (Mag. Theol.),
Wissenschaftlicher Mitarbeiter am Lehrstuhl
für Systematische Theologie II (Ethik),
Friedrich-Alexander-Universität Erlangen-Nürnberg,
Kochstraße 6, 91054 Erlangen, Deutschland

*) Alle Verfasser erklären, dass keinerlei Interessenskonflikte bestehen. Dieser Beitrag entstand im Rahmen des durch das BMBF geförderten Projektes „CwiC - Coping with Certainty" (Förderkennzeichen 01GP1905A).

1) Als „Ungeimpfte" oder „ungeimpfte Personen" werden in diesem Beitrag Personen bezeichnet, die keinen vollständigen Impfschutz gegen Covid-19 besitzen. Ob diese Personen Impfungen gegen andere Krankheiten erhalten haben oder nicht, ist für die Belange dieses Beitrags unerheblich.

2) Mit Verlinkungen zu den weiterführenden Dokumenten: https://www.bundestag.de/dokumente/textarchiv/2021/kw49-de-infektionsschutzgesetz-impfpraevention-870424 (Zugriff am 24.6.2022).

3) Redaktionsnetzwerk Deutschland, Sollten Ungeimpfte höhere Krankenkassenbeiträge zahlen? Scharfe Kritik an *Holetschek*-Vorstoß v. 26.12.2021, im Internet abrufbar unter: https://www.rnd.de/politik/corona-kassenaerzte-chef-gassen-gegen-hoehere-beitraege-fuer-impfunwillige-YXMT5CRHQHA65L7RBKHZYMWZYE.html (Zugriff am: 30.5.2022); *Diringer,* in: Welt am Sonntag v. 10.10.2021, Nr. 41, S. 28/Ressort: Forum.

Vorschlägen einer allgemeinen Erhöhung der Krankenkassenbeiträge für alle Covid-19-ungeimpften Personen, ihrer (Mit-)Beteiligung an ihren Behandlungskosten im Falle einer Covid-19-Erkrankung und die ganz oder teilweise Versagung oder Rückforderung von Krankengeld durch die Krankenkassen[4]. In diesem Beitrag wird aufgezeigt, dass derartige Malusregelungen *de lege lata (1.)* im Recht der GKV nicht umsetzbar sind und dass es darüber hinaus mit Blick auf den Gleichbehandlungsgrundsatz nicht mit geltendem Verfassungsrecht vereinbare wäre, solche Malusregelungen *de lege ferenda (2.)* zu normieren.

### *1. De lege lata*

Zunächst wird erörtert, ob Malusregelungen nach geltendem Recht der GKV angewandt werden könnten. Dazu wird zuerst auf die individuellen Beitragserhöhungen für Ungeimpfte eingegangen (a.) bevor anschließend die Leistungsbeschränkung bei Selbstverschulden nach § 52 SGB V besprochen wird (b.).

#### a) Individuelle Beitragserhöhungen

Die in der politischen Diskussion zuerst vorgeschlagenen Beitragserhöhungen für ungeimpfte Personen lassen sich mit dem Instrumentarium des SGB V nicht umsetzen. Für eine individuell personengebundene Erhöhung des Krankenkassenbeitrags in einer GKV fehlt es bereits an einer Rechtsgrundlage. Der sog. allgemeine Beitragssatz wird stattdessen in § 241 SGB V vom Gesetzgeber festgelegt. Damit soll ganz bewusst eine Beitragsstabilität erwirkt werden, so dass GKV-Mitglieder keinen starken kurzfristigen Schwankungen ausgesetzt sind[5]. Aus diesem Grund hat man sich von früheren Mechanismen der Beitragsveränderungen über Rechtsverordnungen gelöst[6]. Den Krankenkassen wird darüber hinaus über § 242 SGB V die Möglichkeit zur Erhebung *kassen*individueller Zusatzbeiträge gegeben. Diese Zusatzbeiträge werden von *allen* Versicherten einer Krankenkasse *gleichermaßen* erhoben, wenn die Krankenkasse nicht in der Lage ist, ihre Ausgaben über ihre Zuweisungen aus dem Gesundheitsfonds zu bestreiten[7].

#### b) § 52 SGB V

Im Zentrum des juristischen Diskurses um Malusregelungen für ungeimpfte Personen steht zumeist die Leistungsbeschränkung wegen Selbstverschuldens nach § 52 SGB V[8]. Der Grund hierfür liegt in der beachtlichen Rechtsfolge. Die Krankenkasse wird ermächtigt (Abs. 1) bzw. verpflichtet (Abs. 2), Versicherte angemessen an den Behandlungskosten zu beteiligen bzw. das Krankengeld ganz oder teilweise zu versagen oder zurückzufordern. Damit bildet die Norm zum einen eine Ausnahme vom Solidaritätsprinzip, wenn die Solidargemeinschaft nicht für die Kosten der Behandlung aufkommen soll[9]. Zum anderen wird auf die Verursachung der Behandlung Bezug genommen, was eine Ausnahme zum Finalitätsprinzip darstellt[10].

§ 52 Abs. 1 SGB V regelt den Fall, dass sich Versicherte eine Krankheit *vorsätzlich* oder bei einem von ihnen begangenen Verbrechen oder vorsätzlichen Vergehen *zugezogen* haben. Das „Zuziehen" ist nicht legaldefiniert. Insbesondere wird offen gelassen, ob ein aktives Tun hierfür erforderlich ist, oder auch ein Unterlassen, wie das Nicht-impfenlassen, als Anknüpfungspunkt ausreicht[11].

Als Handlung käme das intentionale Treffen mit erkrankten Personen in Betracht. Hierbei würde aber nicht, wie von den oben zitierten Politikern gefordert, an das Ungeimpft-Sein angeknüpft. Die Malusregelung in § 52 Abs. 1 SGB V träfe, wie es auch die Strafnorm aus dem IfSG (§ 75 Abs. 3 i. V. mit § 75 Abs. 1 Nr. 1 IfSG) vorsieht, geimpfte und ungeimpfte Personen gleichermaßen.

Um an das „Ungeimpftsein" anknüpfen zu können, müsste man also tatbestandlich auf ein Unterlassen abstellen. Dies setzt im Allgemeinen jedoch voraus, dass das Unterlassen dem aktiven Tun gleichgestellt werden kann (vgl. § 13 Abs. 1 StGB). Das ist immer dann der Fall, wenn den Betroffenen eine Rechtspflicht zur Vornahme der unterlassenen Handlung trifft[12]. Eine solche könnte sich in einer Impfpflicht, einer Mitwirkungspflicht an einer Heilbehandlung oder allgemeinen Gesunderhaltungspflichten finden. Eine allgemeine, nicht einrichtungsbezogene, Impfpflicht besteht in Deutschland im Hinblick auf SARS-Cov-2 zum Zeitpunkt des Verfassens dieses Aufsatzes nicht. Auch der Verweis auf die Mitwirkungspflicht aus den §§ 63, 65 SGB I kommt für die Gleichstellung von Tun und Unterlassen nicht in Frage. Denn sie setzt eine bereits bestehende Krankheit voraus, an deren Behandlung Versicherte seitens der Krankenkasse aufgefordert werden können, mitzuwirken[13]. Auch allgemeine Gesunderhaltungspflichten sind dem System der GKV fremd. Solche bestehen allenfalls begrenzt auf besondere hoheitliche Rechtsbeziehungen wie z. B. von Soldaten (vgl. § 17a SG).

Selbst wenn man die Vergleichbarkeit von Tun und Unterlassen unterstellte, würde § 52 Abs. 1 SGB V weiterhin noch die (Quasi-)Kausalität zwischen Unterlassen und tatbestandlichem Erfolg notwendig voraussetzen. Damit dieser Kausalzusammenhang bestünde, müsste der tatbestandliche Erfolg beim Hinzudenken der unterlassenen Handlung *mit an Sicherheit grenzender Wahrscheinlichkeit* entfallen (*conditio sine qua non*) und hierauf aufbauend nach der Theorie der wesentlichen Bedingung, die im Sozialversicherungsrecht allgemein anerkannt ist, wesentlich sein. Wobei eine Bedingung dann wesentlich ist, wenn sie nach der Auffassung des praktischen Lebens über eine besondere Beziehung der Ursache zum eingetretenen Erfolg, hier dem Gesundheitsschaden, verfügt[14]. Die Covid-19-Impfung *vermindert* das Risiko, an einem schweren und somit behandlungsbedürftigen wie kostenintensiven Verlauf zu erkranken, sie schließt ihn aber *nicht mit der erforderlichen, an Sicherheit grenzenden Wahrscheinlichkeit* aus. Damit stellt die Covid-Impfung auch keinen *wesentlichen* Grund für die Schwere der Erkrankung dar[15]. Folglich fehlte es auch an der nötigen (Quasi-)Kausalität[16].

4) *Wolff/Zimmermann,* NVwZ 2021, 182, 185 erörtern auch die Möglichkeit die Impfung als Voraussetzung für Sozialleistungen auszugestalten oder steuerliche oder direkte monetäre Vorteile an die Impfung zu knüpfen.

5) *Ulmer,* in: BeckOK SozR, 64. Ed. 1.3.2022, § 241 SGB V, Rdnr. 1.

6) *Peters,* in: KassKomm, 117. EL Dezember 2021, § 241 SGB V, Rdnr. 4.

7) Vgl. § 266 SGB V.

8) S. etwa *Gassner*, ZRP 2022, 2; für die Weiterentwicklung von § 52 SGB V: *Schlegel*, NJW 2021, 2782; Nachteile für Ungeimpfte besprechend: *Duttge*, medstra 2021, 337; unter der Voraussetzung einer Allgemeinen Impfpflicht befürwortend: *Eberbach/Knoepffler*, MedR 2022, 205; schon beim Behandlungsanspruch ansetzend: *Hansen*, GesR 2021, 761; für gesundheitssystemfremd haltend: *Wolff/Zimmermann*, NVwZ 2021, 182, 185.

9) *Heberlein,* in: BeckOK SozR, 64. Ed. 1.3.2022, § 52 SGB V, Rdnr. 17.

10) *Becker/Kingreen,* in: *Becker/Kingreen*, 8. Aufl. 2022, § 11 SGB V, Rdnr. 13.

11) *Heberlein,* in: BeckOK SozR, 64. Ed. 1.3.2022, § 52 SGB V, Rdnr. 6.

12) *Heberlein* in: BeckOK SozR, 64. Ed. 1.3.2022, § 52 SGB V, Rdnr. 6.

13) *Heberlein,* in: BeckOK SozR, 64. Ed. 1.3.2022, § 52 SGB V, Rdnr. 7.

14) BSGE 96, 196, 199 f. m. w. N.

15) Für die gängigen Impfstoffe Comirnaty, Sprikevax und Vaxzevria wird bezüglich der Delta Variante von 90 % Wirksamkeit gegen schwere Verläufe ausgegangen. Die Wirksamkeit der genannten Vakzine bei der Omikron-Variante, ist geringer. Quelle: https://www.rki.de/SharedDocs/FAQ/COVID-Impfen/FAQ_Liste_Wirksamkeit.html (Zugriff am 30.8.2022, mit dem Stand vom 18.8.2022).

16) So auch *Heberlein,* in: BeckOK SozR, 64. Ed. 1.3.2022, § 52 SGB V, Rdnr. 12b.

Zumeist wird die Erfüllung des Tatbestandes auch am Vorsatz scheitern[17]. Dieser setzt in Abgrenzung zur bewussten Fahrlässigkeit Wissen und Wollen der tatbestandserfüllenden Umstände voraus. Wer sich nicht impfen lässt, erwägt dies zumeist aus Vorsicht gegenüber der Neuartigkeit der mRNA-Impfstoffe, ebenfalls neu entwickelter Totimpfstoffe und in diesem Zusammenhang befürchteter Impfnebenwirkungen[18]. Die Möglichkeit der späteren schweren Erkrankung an Covid-19 dürfte in den allermeisten Fällen nicht billigend in Kauf genommen werden, sondern auf den bestmöglichen Ausgang – eine symptomlose Infektion – gehofft werden, womit kein Vorsatz, sondern die nicht tatbestandliche Fahrlässigkeit vorläge[19].

Letztlich würde der Anwendungsbereich der Vorschrift noch dadurch bis zur Marginalität verringert, dass sich der erforderliche Vorsatz in der ersten tatbestandlichen Alternative nicht nur auf die unterlassene Covid-19-Impfung beziehen müsste, sondern darüber hinaus auch noch auf den Kausalverlauf und die Zuziehung der Krankheit[20]. Abschließend ist also festzuhalten, dass das Unterlassen einer Covid-19-Impfung aus vielerlei Gründen nicht unter § 52 Abs. 1 SGB V subsumiert werden kann.

Die Voraussetzungen von § 52 Abs. 2 SGB V sind nicht erfüllt, da die unterlassene Impfung keine medizinisch nicht indizierte ästhetische Operation, Tätowierung oder Piercing darstellt, bei der sich Versicherte eine Krankheit zugezogen haben. Die Aufzählung ist zudem abschließend, sodass der Gesetzgeber keinen Raum für eine analoge Anwendung lässt[21].

Insgesamt ist also festzuhalten, dass das Vorhaben, Ungeimpfte mit einem Malus zu belegen, nicht mit dem geltenden Recht der GKV vereinbar ist[22].

### 2. *De lege ferenda*

Im Folgenden wird aufgezeigt, dass auch eine neu zu implementierende Malusregelung nach den Maßstäben des Gleichheitsgebotes nicht verfassungsgemäß wäre. Hierfür wird zunächst der allgemeine gleichheitsrechtliche Maßstab dargestellt (a), daraufhin werden anhand dreier Fallgruppenvergleiche die in Betracht kommenden (Un-)Gleichbehandlungen und der sich aus ihnen ergebenden Rechtfertigungsmaßstab bestimmt (b) anschließend wird die Frage der verfassungsrechtlichen Rechtfertigung dieser (Un-)Gleichbehandlungen untersucht (c).

#### a) Gleichheitsrechtlicher Maßstab

Auch wenn dem Gesetzgeber grundsätzlich ein Gestaltungsspielraum bei der Ausgestaltung des Leistungskataloges der GKV zusteht[23] und die gesetzlichen Krankenkassen von Verfassung wegen nicht dazu verpflichtet sind, alles zu leisten, was ihre Mittel zulassen[24], ist die gesetzliche Zuordnung der Behandlungskosten von Folgen einer unterlassenen Covid-19-Impfung zur Eigenverantwortung der Versicherten nicht mit dem allgemeinen Gleichheitssatz aus Art. 3 Abs. 1 GG vereinbar.

Nach dem allgemeinen Gleichheitssatz ist wesentlich Gleiches gleich und wesentlich Ungleiches ungleich zu behandeln[25]. Dies gilt gleichermaßen für ungleiche Belastungen als auch für Begünstigungen[26].

Durch Art. 3 Abs. 1 GG werden dem Staat Grenzen gesetzt, wenn dieser Personengruppen innerhalb eines, diese vollständig und abschließend erfassenden Oberbegriffs (*genus proximum*), unterschiedlich behandelt oder umgekehrt, Personengruppen diesem Oberbegriff unterstellt, obwohl sie außerhalb dessen stehen[27]. Diese Grenzen fallen je nach Differenzierungsmerkmal, Regelungsgegenstand und der betroffenen Freiheitsrechte enger oder weiter aus und reichen von einem bloßen Willkürverbot bis hin zu strengen Verhältnismäßigkeitsanforderungen[28].

Zunächst kommt es also darauf an, ob „der Gesetzgeber eine Gruppe von Normadressaten im Vergleich zu einer anderen […] anders behandelt, obwohl zwischen beiden Gruppen keine Unterschiede von solcher Art und von solchem Gewicht bestehen, dass sie die ungleiche Behandlung rechtfertigen“[29].

#### b) Fallgruppenvergleiche

Vorliegend kommen innerhalb des Systems der GKV drei (Un-)Gleichbehandlungen in Betracht. Vergleichsgruppen in der Privaten Krankenversicherung werden aufgrund der Strukturunterschiede zwischen den Systemen und des hier verfolgten Schwerpunktes bewusst ausgeblendet.

Nach der zentralen Idee der Malusregelungen würden Ungeimpfte mit Fällen einer vorsätzlichen Erkrankung nach § 52 SGB V gleichgestellt (aa.). Gleichzeitig würden schwer an Covid-19 erkrankte Ungeimpfte danach anders behandelt als Geimpfte, die ebenfalls schwer an Covid-19 erkrankt sind (bb.) und zwar auch dann, wenn Geimpfte das Risiko eines schweren Krankheitsverlaufs durch ihre Lebensführung aktiv erhöht haben (cc.).

##### aa) Erste Fallgruppe – Fälle des § 52 SGB V

Die Fälle des § 52 SGB V unterscheiden sich wesentlich von denen der unterlassenen Covid-19-Impfung, insofern dass bei ersteren ein eigenes Risiko aktiv geschaffen wird, bzw. sie sich „aus eigenem Entschluss gesundheitlichen Risiken aussetzen“[30], während bei Letzteren keine solche Risikosteigerung durch den Betroffenen stattfindet[31]. Denn in letzterem Fall wurde das allgemeine Lebensrisiko durch das erstmalige Auftreten der Krankheit für *alle* Menschen gleichermaßen – ohne deren Zutun – erhöht. Folglich besteht ein Unterschied zwischen der Gruppe der Personen, die ihre Erkrankung, wie in § 52 SGB V normiert, selbstverschuldet haben und jenen, deren Risiko durch äußere Umstände, die nicht in ihrer Verantwortung lagen, erhöht wurde.

Weiterhin unterscheiden sich die beiden Konstellationen darin, ob die Erkrankung auf eine konkrete Ursache zurückgeführt werden kann oder nicht. Denn die Covid-19-Erkrankung kann *nicht als unmittelbare Folge* der unter-

17) Ebenfalls *Heberlein,* in: BeckOK SozR, 64. Ed. 1.3.2022, § 52 SGB V, Rdnr. 12b; allgemeiner auch schon *Huster*, Ethik Med 2010, 289, 292 f.
18) Zu den Beweggründen gegen eine Covid-19-Impfung: *Troiano/Nardi*, Public Health 2021, 245.
19) So auch schon *Gassner*, ZRP 2022, 4.
20) Dies gilt ausdrücklich nicht für die übrigen Varianten, vgl. BT-Dr. 11/2237, zu § 51, S. 182.
21) Vgl. SG Mainz, Gerichtsbescheid v. 21.9.2015 – S 3 KR 558/14; so auch: *Heberlein,* in: BeckOK SozR, 64. Ed. 1.3.2022, § 52 SGB V, Rdnr. 21; *Reyels,* in: jurisPK-SGB V, 4. Aufl., § 52 SGB V, Rdnr. 113.
22) Im Ergebnis auch *Bockholdt,* in: *Schlegel/Meßling/Bockholdt*, Covid-19 Corona-Gesetzgebung, § 11, Rdnrn. 10–13; s. hierzu auch die Antwort des Parlamentarischen Staatssekretärs im BMG Dr. *Gebhart* im Deutschen Bundestag v. 28.12.2020, BT-Dr. 19/25571, Frage Nr. 154, S. 112.
23) Vgl. BVerfGE 115, 25, 38 ff.
24) Vgl. BVerfGE 115, 25, 46 m.w.N.
25) Vgl. BVerfGE 1, 14, 52.
26) Vgl. BVerfGE 79, 1, 17; BVerfGE 126, 400, 416; BVerfGE 129, 49, 68.
27) Vgl. *Heun*, in: *Dreier*, GG-Kommentar, Art. 3, Rdnrn. 18, 24.
28) Vgl. BVerfGE 75, 108, 157; BVerfGE 107, 27, 45 f.; BVerfGE 126, 400, 416 m.w.N.
29) St. Rspr., vgl. BVerfGE 112, 50, 67; BVerfGE 117, 316, 325.
30) BT-Dr. 16/3100, Zu Nummer 31 (§ 52), S. 108; zur Abgrenzbarkeit der Verhaltensweisen insbesondere die Vorbemerkung und Fragen 1–5 zur kleinen Anfrage BT-Dr. 17/9213, S. 2 f., 5; BT-Dr. 17/8509, Frage 60, S. 36.
31) So auch *Heberlein,* in: BeckOK, 64. Ed. 1.3.2022, § 52 SGB V, Rdnr. 12b.

lassenen Impfung gesehen werden und ist auch *nicht leicht abgrenzbar* von anderen Ursachen, wie genetischen oder umweltbedingten Faktoren[32].

Wenn der Gesetzgeber argumentiert, dass die Verhaltensweisen der Versicherten, wie sie § 52 SGB V umschreibt, unmittelbare Folgen hätten und *leicht abgegrenzt* werden könnten[33], was beides im Hinblick auf Verhaltensweisen der allgemeinen Lebensführung, die abstrakt risikobehaftet sind, wie etwa das Rauchen oder eine Übergewicht begünstigende Ernährung, *nicht* gegeben sei, weshalb diese von § 52 SGB V ausgenommen sind[34], dann muss gleiches auch für die unterlassene Impfung gelten.

bb) Zweite Fallgruppe – Geimpfte Personen

Weiterhin bestünde eine Ungleichbehandlung zwischen geimpften und ungeimpften Personen, die jeweils einen schweren Verlauf von Covid-19 erleiden. Während Erstere die Behandlungskosten durch die Solidargemeinschaft finanziert bekämen, sollen Letztere bei Einführung von Malusregelungen, zumindest einen Teil ihrer Behandlungskosten selbst tragen müssen. Als Differenzierungsmerkmal dient hier alleinig der Impfstatus. Diesem Differenzierungsmerkmal liegt die Logik inne, dass die Impfung auch im Einzelfall einen solch signifikanten Einfluss auf die Schwere einer Covid-19-Erkrankung hat, dass sie auch eine Unterscheidung hinsichtlich der Frage erlaubt, wer welche Anteile an den Behandlungskosten zu tragen hat. Ob diese Logik überzeugt, also ob dieses Merkmal einen „wesentlichen" Unterschied der Vergleichsgruppen kennzeichnet, ist eine Frage der Rechtfertigung[35].

cc) Dritte Fallgruppe – Risikosteigernde Verhaltensweisen der allgemeinen Lebensführung

Weiterhin bestünde bei der Umsetzung einer solchen Malusregelung eine Ungleichbehandlung zwischen *ungeimpften* Personen mit schwerem Covid-19-Verlauf und *geimpften* Personen mit schwerem Covid-19-Verlauf, die ihr Risiko einen schweren Verlauf zu erleiden durch eine ungesunde Lebensführung, wie z.B. durch das Rauchen oder durch eine Übergewicht-begünstigende Ernährung negativ beeinflusst haben.

Wenn man sich auf den Standpunkt stellt, dass das nicht-impfen-Lassen mit großer Sicherheit in einer behandlungsbedürftigen Erkrankung resultieren wird, dann erweist es sich als widersprüchlich, wenn einerseits die Impfverweigerung als sicher gefahrenerhöhend sanktioniert wird, andere Verhaltensweisen, die tatsächlich einen schweren Krankheitsverlauf mit Covid-19 aktiv begünstigen, jedoch nicht[36]. Dieses Missverhältnis ist umso gravierender, als dass z.B. Raucher und ungesund ernährte Personen ihr allgemeines Lebensrisiko durch ein neues Risiko gesteigert haben[37], wohingegen ungeimpfte Personen kein neues Risiko geschaffen haben[38]. Im Sinne des Erst-Recht-Schlusses müssen Leistungsbeschränkungen folglich sogar aus zwei Gründen bei Letzteren unterbleiben, wenn dies bei Ersteren auch der Fall ist.

c) Verfassungsrechtliche Rechtfertigung

Diese (Un-)Gleichbehandlungen können gleichwohl verfassungsrechtlich gerechtfertigt sein. Hierfür ist der Maßstab der Rechtfertigung zu bestimmen. Dieser hängt zunächst davon ab, ob das Differenzierungskriterium für die Betroffenen beeinflussbar ist oder in deren Person verankert ist. Bei ersterem werden zunächst geringe Rechtfertigungsanforderungen gestellt[39]. Im Fall von Malusregelungen für ungeimpfte Personen würde nicht an ein Differenzierungsmerkmal angeknüpft, das unveränderbar in der Persönlichkeit des Betroffenen liegt – das Merkmal des (Un-)Geimpft-Seins ist (mittlerweile in Deutschland) für jeden Einzelnen frei veränderbar. Ein strengerer, an Verhältnismäßigkeitsgesichtspunkten orientierter Maßstab kann sich gleichwohl ergeben, wenn mit der Ungleichbehandlung die Wahrnehmung von hochwertigen Freiheitsrechten erheblich beeinflusst wird[40]. Wenn der Gesetzgeber an das Ungeimpft-Sein negative Konsequenzen knüpft, müssten die Versicherten, um der intendierten Aufforderung des Normgebers zu folgen, die unterlassene Impfung vornehmen. Hierdurch ist die körperliche Unversehrtheit der Versicherten aus Art 2 Abs. 2 S. 1 2. Alt. GG betroffen. Denn das Impfen setzt einerseits zur Erstimpfung notwendig die Injektion des Vakzins mit einer Spritze voraus und löst andererseits fast schon regelgerecht vorübergehende Impfnebenwirkungen (und selten anhaltende Impfschäden) aus.

Direkt betroffen von einer Norm, die bei unterlassener Impfung die Leistungsbeschränkung anordnet, wäre die allgemeine Handlungsfreiheit aus Art. 2 Abs. 1 GG. Schließlich können sich auch aus den Eigenarten des betroffenen Regelungsbereichs strenge Rechtfertigungsanforderungen an die (Un-)Gleichbehandlungen ergeben[41]. Der Regelungsbereich ist vorliegend durch die Eigenarten der solidarischen Krankenversicherung, namentlich durch die final gewährte Kostenübernahme für Krankenbehandlungen durch die gesetzlichen Krankenkassen geprägt. Dieser Regelungsbereich zeichnet sich besonders durch einen gleichberechtigten Zugang innerhalb der Versicherungsgemeinschaft und durch Blindheit hinsichtlich der Ursachen der behandlungsbedürftigen Krankheit aus[42]. Die intendierten Regelungen laufen auf eine Ungleichbehandlung von Personengruppen, die durch die Versicherungspflicht in der Krankenversicherung zwangsweise zusammengefasst sind, was bereits regelmäßig zu strengeren Rechtfertigungsanforderungen führt[43]. Zudem steht die Kostenübernahme einer besonders schweren Erkrankung mit potenziell langen Folgen[44] zur Diskussion, was für Versicherte eine empfindliche, hinsichtlich der Behandlungsaufnahme, abschreckende Wirkung auch dann noch entfalten könnte, wenn man die aus dem Naturalleistungsprinzip folgende Vorleistungspflichten der Krankenkassen berücksichtigt[45]. All dies spricht für einen erhöhten Rechtfertigungsmaßstab für die angedachten Malusregelungen. Folglich ist der strengere, am Grundsatz der Verhältnismäßigkeit orientier-

32) Zur Quasikausalität s. oben I. 1. b.
33) Vgl. BT-Dr. 17/8509, Frage 60, S. 36; BT-Dr. 17/9213, S. 2.
34) Vgl. BT-Dr. 17/8509, Frage 60, S. 36.
35) Das Kriterium der Wesentlichkeit der (Un-)gleichbehandlung problematisierend: *Heun*, in: *Dreier*, GG-Kommentar, Art. 3, Rdnr. 25.
36) Vgl. zu einer solchen Wertung im Kontext von § 52 Abs. 2 SGB V bereits *Lang*, in: *Becker/Kingreen*, 8. Aufl. 2022, § 52 SGB V, Rdnr. 8a; zu den weiteren Risikofaktoren vgl. die Informationen des RKI https://www.rki.de/DE/Content/InfAZ/N/Neuartiges_Coronavirus/Risikogruppen.html (Zugriff am: 29. 8. 2022).
37) *Shirvaliloo,* Epigenomics 14 (3), 153; *Clift/von Ende/Piu,* Thorax 2022, 77: 65.
38) Vgl. *Heberlein,* in: BeckOK SozR, 64. Ed. 1. 3. 2022, § 52 SGB V, Rdnr. 12b.
39) Vgl. *Heun*, in: *Dreier*, GG-Kommentar, 3. Aufl. 2013, Art. 3, Rdnr. 38.
40) Vgl. BVerfGE 88, 87, 96.
41) Mit den Worten des BVerfG: „Der normative Gehalt der Gleichheitsbindung erfährt daher seine Präzisierung jeweils im Hinblick auf die Eigenart des zu regelnden Sachbereichs." etwa in BVerfGE 75, 108; BVerfGE 93, 319; vgl. auch *Heun,* in: *Dreier,* GG-Kommentar, 3. Aufl. 2013, Art. 3, Rdnrn. 21 ff.
42) Vgl. BSGE 59, 119; *Vossen,* in: *Krauskopf,* Soziale Krankenversicherung, 113. EL Dezember 2021, § 52 SGB V, Rdnr. 2; *Ebsen/Wallrabenstein*, in: *Ruland/Becker/Axer*, Sozialrechtshandbuch, § 52 SGB V, Rdnr. 76; *Prehn*, NZS 2010, 260, 263.
43) BVerfGE 92, 53; BVerfG, BVerfGE 89, 365; *Kischel,* in: BeckOK GG, 51. Ed. 15. 5. 2022, Art. 3, Rdnr. 173.
44) S. überblicksweise: *Funke-Chambour,* Praxis 2021, 377.
45) Das Naturalleistungsprinzip als argumentative Stütze von § 52 SGB V ansehend: BSG, NJW 2020, 708.

te Maßstab anzuwenden, um zu prüfen, ob der Impfstatus einen Unterschied von solcher Art und solchem Gewicht darstellt, dass er die aufgezeigten (Un-)Gleichbehandlungen rechtfertigen kann.

Mit Blick auf die angestrebte Rechtsfolge, ließe sich die Kosteneinsparung der gesetzlichen Krankenkassen als grundsätzlich legitimes Ziel[46] der Maßnahme benennen. Ein weiteres Ziel läge im bewussten Schutz der Solidargemeinschaft vor der Kostenübernahme von Versicherungsfällen, die von einer besonderen Eigenverantwortlichkeit der Versicherten geprägt sind und bei denen es ungebührlich erschiene, die Solidargemeinschaft an diesen Kosten zu beteiligen.

Die Leistungen der Krankenkassen zu beschränken und damit den Versicherten die Behandlungskosten (teilweise) aufzuerlegen, wäre auch ein geeignetes Mittel, um beide Ziele zu erreichen, denn das Gesundheitssystem würde hierdurch finanziell entlastet. Prinzipiell kann auf diese Weise auch eine Belastung der Krankenkassen mit Behandlungskosten verhindert werden, die eher der Eigenverantwortlichkeit der Versicherten entspringen[47]. Es besteht weiterhin auch eine starke gesetzgeberische Einschätzungsprärogative im regulativen Umfeld von § 52 SGB V[48], welche dem Gesetzgeber viel Freiheit bei der Auswahl des mildesten unter den gleich geeigneten Mitteln der Zweckerfüllung belässt[49]. Letztlich müssten die Mittel der (Un-)Gleichbehandlung, mit denen das legitime Ziel verfolgt wird, auch angemessen sein, das heißt, dass der Nutzen der Maßnahme im Verhältnis stehen muss, zu den Beeinträchtigungen, die die Maßnahme verursacht. Grundsätzlich ist die finanzielle Stabilität der gesetzlichen Krankenkassen ein wichtiger Aspekt für dessen Fortbestand. Denn dieser bildet die Grundlage dafür, dass der Staat seinen verfassungsrechtlichen Auftrag zur Gesundheitsversorgung erfüllt[50].

Dem stehen die erwartbaren negativen Folgen gegenüber, die über die Leistungsverweigerung im Einzelfall hinausreichen. Hier sind gleich mehrere zu nennen. Aufgrund der Ausgrenzung einer Gruppe und der hier verwendeten Ausgrenzungsrhetorik, wäre im schlimmsten Fall die weitere Spaltung der Gesellschaft zu befürchten[51]. Beim Versuch, die vermeintlich fehlende, verhaltensbezogene Solidarität der ungeimpften Personen zu sanktionieren, würde ein anderer Aspekt der Solidarität untergraben, nämlich der zwischen Gesunden und Kranken: Die GKV beruht als Versicherung auf dem Prinzip, dass erkrankte Versicherte die Behandlungskosten faktisch durch die Beiträge der gesunden Versicherten (mit) abdecken – insofern verhalten sich die gesunden Versicherten über das Instrument der Versicherung solidarisch mit den Kranken[52]. Durch die Malusregelungen würde sich nun diese Solidarität der Gesunden nur noch auf einen Teil der Kranken, nämlich den der Geimpften beziehen, und die Ungeimpften wären von dieser Solidarität ausgeschlossen. Das Prinzip der Solidarität der Gesunden mit den Kranken wäre somit partiell aufgehoben. Die Gruppe der Versicherten würde hinsichtlich ihrer finanziellen Solidarität zueinander aufgespalten – und zwar unabhängig davon, ob dies von den gesunden Versicherten gewollt ist[53]. Dieser induzierte Verlust solidarischer finanzieller Absicherung wiegt für die an Covid-19 Erkrankten besonders schwer, wo es doch das Ansinnen der solidarischen GKV ist, erkrankten Personen die zusätzliche Bürde finanzieller Belastungen zu ersparen (Stichwort Sachleistungsprinzip)[54]. In konkreten Zahlen können das bei einem durchschnittlichen Behandlungsumfang und durchschnittlicher Liegezeit auf einer Covid-19-Intensivstation zwischen 30.000 und 40.000 € sein[55].

Die Unterscheidung in der Gruppe der an Covid-19 Erkrankten anhand der Impfung fußt nun auf einer gewissen epistemischen Autorität der Wirksamkeitsstudien zu den Impfstoffen. Weil die (schwere) Erkrankung durch die Impfung mit hoher Wahrscheinlichkeit verhindert werden könne, sei eine Leistungsverweigerung bei einer bewussten Weigerung der Risikoreduktion gerechtfertigt. Eine solche Argumentation birgt jedoch die Gefahr, dass es zu Dammbruch-Effekten kommen, die weitere Ausschlüsse von der Solidargemeinschaft nach sich ziehen könnten[56]. Wenn der Gesetzgeber mit einer Malusregelung erstmals Verhaltensweisen von unterlassener Risikoreduktion sanktioniert, dann ist die Hürde geringer, diese Unterscheidungsweise auch auf andere Bereiche zu übertragen. So lassen sich etwa auch viele Gesundheitsrisiken mittels Gendiagnostik und der Prädiktion durch KI-Big-Data-Anwendungen frühzeitig erkennen und reduzieren oder gar verhindern[57]. Der Verweis, dass gewisse durch Gendiagnostik oder KI-Prädiktionen entdeckte Gesundheitsrisiken nicht beeinflusst werden können, muss nicht notwendigerweise Schutz vor einem etwaigen Selbstbehalt bieten, denn auch an diesen Risikoprognosen lässt sich das eigenen gesundheitsbezogene Verhalten entsprechend ausrichten[58]. Noch stärker streitet ein solches epistemisches Argument dafür, weiteres gefahrerhöhendes Verhalten vom Leistungsspektrum der GKV auszuschließen[59]. Es ist gesellschaftlich nicht

46) S. etwa:BVerfG, NJW 2001, 1779, 1780.
47) Vgl. zu letzterem BSG, NZS 2020, 58, 61.
48) Vgl. BSG, NJW 2020, 708.
49) Zur nicht unerheblichen Frage, ob Bonusprogramme oder eine stärkere Aufklärungskampagne besser geeignet erscheinen vgl. *Heberlein,* in: BeckOK SozR, 64. Ed. 1. 3. 2022, § 52 SGB V, Rdnrn. 12c f. und *Wolff/Zimmermann*, NVwZ 2021, 182, 185; grundlegend schon *Huster*, Ethik Med 2010, 289, 298. Beides wirkt sich gleichwohl nur mittelbar durch die Erhöhung der Impfquote und die entsprechend erwartbare Reduktion von Versicherungsfällen auf die finanzielle Ausstattung der Krankenkassen aus.
50) Vgl. insbesondere mit Blick auf die Beitragsstabilität: BVerfGE 68, 193, 218; BVerfGE 103, 172, 184 f.; näher dazu: *Quaas/Zuck/Clemens*, Medizinrecht, § 2 Verfassungs- und europarechtliche Vorgaben, Rdnrn. 8, 83 ff.
51) *Augsberg*, DLF Interview v. 1. 1. 2022, 17:47 Uhr, im Internet abrufbar unter: Impfpflicht und Ausgrenzungsrhetorik – Steffen Augsberg im Gespräch | deutschlandfunk.de (Zugriff am: 27. 6. 2022).
52) Bezogen auf die allgemeinen Auswirkungen von Zuzahlungen auf Solidarität zwischen Gesunden und Kranken auch: *Süß*, Die Eigenverantwortung gesetzlich Krankenversicherter unter besonderer Berücksichtigung wunscherfüllender Medizin, S. 172.
53) Vgl. *Hörnle*, Ethik Med 2022, Im Internet abrufbar unter: https://doi.org/10.1007/s00481-022-00716-8 (Zugriff am 24. 8. 2022), die über die negativen Reaktionen – auch der geimpften Leserschaft – auf ihrer Stellungnahme pro Berücksichtigung des Impfstatus im Kontext der Covid-19-Pandemie (2021) berichtet.
54) Vgl. *Joussen*, in: BeckOK SozR, 64. Ed. 1. 3. 2022, § 2 SGB V, Rdnr. 6.
55) Im Internet abrufbar unter: https://www.aerztezeitung.de/Wirtschaft/Was-kosten-Corona-Intensivpatienten-425104.html (Zugriff am 24. 6. 2022).
56) Differenzierend zwischen der Priorisierung von Geimpften bei der unmittelbaren Versorgung mit Gesundheitsleistungen in der Extremsituation einer Triage und der nachfolgenden Frage der Kostenübernahme: *Hörnle*, Ethik Med 2022, Im Internet abrufbar unter: https://doi.org/10.1007/s00481-022-00716-8 (Zugriff am 24. 8. 2022).
57) Prophylaktische Operationen, wie die Mastektomie im Fall Angelina Jolies andenkend: *König/Junge*, GuP 2015, 132, 136, der hinsichtlich der Strafbarkeit gesundheitswidrigen Verhaltens verweist auf: *Höffler*, in: *Wienke/Dierks/Janke,* Rechtsfragen der Personalisierten Medizin, S. 63; zu den Auswirkungen von KI-Prädiktionen auf die GKV: *von Ulmenstein/Tretter/Ehrlich/Lauppert von Peharnik*, Front. Artif. Intell. 5:913093, doi: 10.3389/frai.2022.913093.
58) Vgl. insbesondere *Paul*, Ethik Med 2010, 191, 197, der das Beispiel eines fortgesetzten Zigarettenkonsums trotz festgestellter genetischer Prädisposition für Lungenkrebs anführt.
59) Dieses Problem zeigt sich bereits bei § 52 Abs. 2 SGB V, vgl. *Lang* in: *Becker/Kingreen*, 8. Aufl. 2022, § 52 SGB V, Rdnrn. 8 ff. m. w. N.

unbedingt wünschenswert, dass Versicherte alle möglichen präventiven Maßnahmen ergreifen müssen, um der Leistungsbeschränkung durch die Krankenkassen zu entgehen[60]. Jedenfalls ist es kein ausdrücklich dem Recht der GKV entspringender Automatismus, welches zudem gar nicht auf die mit den Malusregelungen zusammenhängenden ethischen Bewertungen von gesundheitsbezogenem Verhalten ausgerichtet ist[61]. Anders ausgedrückt wird in der GKV Solidarität im Krankheitsfall nicht epistemisch vorgefunden, sondern normativ geformt. Ohne die epistemische Autorität der Wirksamkeitsstudien verbleiben jedoch kaum noch nennenswerte Gründe, um die beschriebenen (Un-)Gleichbehandlungen zu rechtfertigen.

Zu der beschriebenen (Un-)gleichbehandlung innerhalb der Versichertengemeinschaft der GKV tritt die ohnehin komplexe rechtliche Wechselbeziehung zwischen GKV und PKV. Ohne dass hierauf im Detail eingegangen werden kann, kann es doch als problematisch angesehen werden, wenn in der PKV die Behandlungskosten für Ungeimpfte bei einer Covid-19-Erkrankung erstattet würden[62], Ungeimpfte in der GKV hingegen obendrein mit einem Malus belegt würden.

Weiterhin besteht an vielen Stellen noch Unklarheit darüber, wie der Vorschlag von Malusregelungen in praktischer Hinsicht umzusetzen ist: Was als angemessene Kostenbeteiligung[63] anzusehen ist, wie sich der Nachweis des Impfstatus gegenüber der GKV ohne ein zentrales Impfregister umsetzen ließe[64], wer per Definition als geimpft gilt (z.B. Erst-und Zweitgeimpfte, Geboosterte, Genesene), wie die von den Impfempfehlungen ausgenommene Personenkreise (z.B. Allergiker, kleine Kinder und Schwangere im ersten Trimenon) und beitragsfrei Familienversicherte einzuordnen sind[65]. Um diese Vorfragen zu klären und Umsetzungshindernisse zu beseitigen, müssten wohl erhebliche personelle und finanzielle Ressourcen eingesetzt werden, die sich freilich den anvisierten Kostenersparnissen durch die Malusregelungen gegenüberstellen lassen und diese in Frage stellen.

Insgesamt zeigt sich damit, dass die in den Malusregelungen enthaltene Ungleichbehandlung negative Folgen aufweist, die weiter reichen, als sie zur Erreichung des legitimen Ziels beitragen würden. Dieses regulative Mittel erscheint insbesondere angesichts seiner Blindheit für die im Spannungsfeld von Solidarität und Eigenverantwortung existierenden Problemkontexte in einem unangemessenen Ungleichgewicht, wenn es sich auf die infektiologische Gewissheitsprognose der Impfung verlässt. Es ist daher verfassungsrechtlich nicht gerechtfertigt.

## II. Fazit und Ausblick

Ausgehend vom konkreten politischen Vorschlag, Malusregelungen für Covid-19 ungeimpfte Personen zu implementieren, setzte sich dieser Beitrag mit den Fragen auseinander, ob *derartige Malusregelungen im gegenwärtigen Recht der GKV vereinbar wären und ob es andernfalls verfassungsrechtlich zulässig wäre, solche Malusregelungen rechtlich zu normieren*. Der Beitrag hat aufgezeigt, dass Malusregelungen *de lege lata* nicht umsetzbar sind, weil es einerseits für individuelle Beitragserhöhungen keine Rechtsgrundlage gibt und andererseits die Voraussetzungen für die Leistungsbeschränkung wegen Selbstverschuldens nach § 52 SGB V nicht erfüllt sind. Auch mit Blick auf die Rechtslage *de lege ferenda*, kann auf kein anderes Ergebnis geschlossen werden, denn neu geschaffene Malusregelungen würden gegen den Gleichheitsgrundsatz verstoßen. Ein Gesetzesentwurf, der sich auf die sehr unkonkret gehaltenen Vorschläge *Boris Palmers* und *Klaus Holetscheks* stützt, erwiese sich insofern als nicht mit geltendem Verfassungsrecht vereinbar.

Der Aufsatz liefert einen Beitrag zur rechtlichen Diskussion um Malusregelungen für ungeimpfte Personen. Gleichzeitig besitzen die enthaltenen Überlegungen über den konkreten Kontext hinaus Relevanz. Denn sie liefern Impulse zur Bestimmung des Zusammenhangs von Eigenverantwortung und Solidarität – und damit für Fragen, die in Zukunft, mit neuen Möglichkeiten einer KI-basierten, prognostischen Medizin und schwindenden medizinischen Ungewissheiten, immer häufiger aufkommen werden. Die Normerwartung, KI-Prädiktion als Präventionsinstrument in Anspruch zu nehmen, könnte sich in diesem Zusammenhang als Triebfeder der Entsolidarisierung erweisen. Denn im vorliegenden Kontext hat schon die Möglichkeit der punktuellen, auf eine Krankheit bezogenen Risikoreduktion ausgereicht, um entsolidarisierende Tendenzen zum Vorschein zu bringen. Überträgt man dies auf eine Vielzahl von Krankheiten und ein höheres Maß an Gewissheit, dann wird deutlich, welches Problempotential in neuen Gewissheiten über Kausalzusammenhänge zwischen gesundheitsschädigendem Verhalten und Erkrankung bzw. zwischen unterlassener Prävention und Erkrankung für das solidarische Gesundheitssystem steckt.

**Open Access.** Dieser Artikel wird unter der Creative Commons Namensnennung 4.0 International Lizenz veröffentlicht, welche die Nutzung, Vervielfältigung, Bearbeitung, Verbreitung und Wiedergabe in jeglichem Medium und Format erlaubt, sofern Sie den/die ursprünglichen Autor(en) und die Quelle ordnungsgemäß nennen, einen Link zur Creative Commons Lizenz beifügen und angeben, ob Änderungen vorgenommen wurden.
Die in diesem Artikel enthaltenen Bilder und sonstiges Drittmaterial unterliegen ebenfalls der genannten Creative Commons Lizenz, sofern sich aus der Abbildungslegende nichts anderes ergibt. Sofern das betreffende Material nicht unter der genannten Creative Commons Lizenz steht und die betreffende Handlung nicht nach gesetzlichen Vorschriften erlaubt ist, ist für die oben aufgeführten Weiterverwendungen des Materials die Einwilligung des jeweiligen Rechteinhabers einzuholen.
Weitere Details zur Lizenz entnehmen Sie bitte der Lizenzinformation auf http://creativecommons.org/licenses/by/4.0/deed.de.

60) So schon *Eberbach,* MedR 2010, 756, 763; in einer Dystopie verarbeitend: *Zeh,* Corpus Delicti Ein Prozess, 2009.
61) So, wenn auch in anderem Kontext *Hörnle*, Ethik Med 2022, Im Internet abrufbar unter: https://doi.org/10.1007/s00481-022-00716-8 (Zugriff am 24. 8. 2022).
62) Vom Verband der Privaten Krankenversicherungen sei dies laut Recherche des SWR jedenfalls im September 2021 nicht vorgesehen, auch wenn einzelne Versicherungen Tendenzen hierzu zeigten, https://www.swr.de/swraktuell/baden-wuerttemberg/braucht-es-krankenkassentarife-fuer-ungeimpfte-100.html (Zugriff am: 30. 8. 2022).
63) Zum Ausgleich sozialer Härten, als die Verhältnismäßigkeit wahrendes Konzept vgl. *Süß*, Die Eigenverantwortung gesetzlich Krankenversicherter unter besonderer Berücksichtigung wunscherfüllender Medizin, S. 172 u. a. m.Verw. auf *Hänlein*, SGB 2003, 301, 309, mit Anmerkungen zur generellen Schutzbedürftigkeit der Versicherten.
64) Für die Schaffung eines Impfregisters: *Gassner*, ZRP 2022, 5.
65) Vgl. hierzu die Empfehlungen des RKI in Schwangerschaft, Stillzeit und bei Kinderwunsch https://www.rki.de/SharedDocs/FAQ/COVID-Impfen/FAQ_Liste_Impfung_Schwangere_Stillende.html (Zugriff am: 29. 8. 2022 und die Empfehlungen der STIKO hinsichtlich der Altersgruppe 0–5 Jahre und 5–12 Jahre https://www.rki.de/SharedDocs/FAQ/COVID-Impfen/FAQ_Liste_Impfung_Kinder_Jugendliche.html (Zugriff am: 29. 8. 2022); ergänzend zur Familienversicherung die Ausführungen bei *Gassner*, ZRP 2022, 1, 3.

Funding

Open access funding enabled and organized by Projekt DEAL.

